# Supplementary material for: Biology, Systematics, Microbiome, Pathogen Transmission and Control of Chiggers (Acari: Trombiculidae, Leeuwenhoekiidae) with Emphasis on the United States
Source: Int J Environ Res Public Health. 2022 Nov 17;19(22):15147. doi: 10.3390/ijerph192215147 (PMC9690316; doi:10.3390/ijerph192215147)
Supplement: Supplementary file 1 [file ijerph-19-15147-s001.zip › ijerph-1914582-supplementary.pdf]

Table S1. Search strategy used for Figure 1 was #1 AND #2 from Web of Science Core Collection.

Concept	Search	**Search strategy**
Concept 1 (Chiggers)	#1	(((((TS[1]= ("Larval mite*")) OR TS=("larval trombiculid mite*")) OR TS=("Chigger*")) OR TS=("Harvest Mite*")) OR TS=("berry bug*")) OR TS=("trombiculid mite*")
Concept 2 (United States)	#2	((TS=("New world")) OR TS=("U.S.A.")) OR TS=("U.S.")) OR TS=("US")) OR TS=("USA")) OR TS=("United States")) OR TS=("Alabama")) OR TS=("indiana")) OR TS=("Alaska")) OR TS=("Arizona")) OR TS=("Arkansas")) OR TS=("California")) OR TS=("Colorado")) OR TS=("Connecticut")) OR TS=("Delaware")) OR TS=("Florida")) OR TS=("Georgia")) OR TS=("Hawaii")) OR TS=("Idaho")) OR TS=("Illinois")) OR TS=("Iowa")) OR TS=("Kansas")) OR TS=("Kentucky")) OR TS=("Louisiana")) OR TS=("Maine")) OR TS=(Maryland)) OR TS=(Massachusetts)) OR TS=(Michigan)) OR TS=(Minnesota)) OR TS=(Mississippi)) OR TS=(Missouri)) OR TS=(Montana)) OR TS=(Nebraska)) OR TS=(Nevada)) OR TS=("New Hampshire")) OR TS=("New Jersey")) OR TS=("New Mexico")) OR TS=("New York")) OR TS=("North Carolina")) OR TS=("North Dakota")) OR TS=(Ohio)) OR TS=(Oklahoma)) OR TS=(Oregon)) OR TS=(Pennsylvania)) OR TS=("Rhode Island")) OR TS=("South Carolina")) OR TS=("South Dakota")) OR TS=(Tennessee)) OR TS=(Texas)) OR TS=(Utah)) OR TS=(Vermont)) OR TS=(Virginia)) OR TS=(Washington)) OR TS=("West Virginia")) OR TS=(Wisconsin)) OR TS=(Wyoming)) OR TS=("Puerto Rico")

[1]TS: Topic (Searches title, abstract, author keywords, and Keywords Plus)

Table S2. Summary of publications of chiggers in the US. Publications were searched through the Web of Science on May 3, 2022 (search strategy provided in Table S1.).

References	**Hosts**	**States**	**Chigger species**	**Summary of paper**
Ewing and Hartzell 1918 [131]	Humans, Locust, House fly	Mississippi, Omaha, Nebraska, Oregon, Missouri, New York, Illinois and Iowa	"The American chigger mite", "the irritating chigger mite", *Euthrombidium trigonum*, *Thrombidium muscarum*	Reviewed of chiggers reported from Europe and the US
Ewing 1923 [132]	Humans	NA*	*Trombicula tlalzahuatl* (Murray)	Taxonomy and distribution of common North American chigger
Ewing 1925a [133]	NA	NA	*Trombicula irritans*	Taxonomy: Provided a list of synonymies of *Trombicula irritans*; Provided a key to the American chigger species
Ewing 1925b [134]	NA	NA	*Trombicula irritans*	Report of the adult stage of common North American chigger
Ewing 1929 [135]	Field sparrow, Towhee, Prothonotary warbler, Maryland yellowthroat, Redstart, Carolina wren, Blue-gray gnatcatcher	Virginia and North Carolina	*Trombicula irritans*	Survey of chiggers on birds
Ewing 1931 [136]	NA	NA	45 species	Chigger taxonomy: provided a catalog of 45 species of chiggers of New World and a key to nine genera
Ewing 1938 [137]	NA	NA	*Euschongastia*, *Eutrombicula*, *Hemitrombicula*, *Gateria*	Taxonomy and Host relationships: Provided a key to the genera of chiggers with descriptions of new genera and species host relationships
Ewing 1942 [138]	NA	NA	*Schongastia lynni*, *Trombicula rohweri*, *Trombicula californica*, *Comatacarus occidentalis*, *Comatacarus americanus*, *Acomatacarus arizonensis* and *Walchia americ*	Taxonomy: Remarks on the taxonomy of some American chiggers including the descriptions of new genera and species

Ewing 1943 [139]	NA	NA	*Acariscus*	Taxonomy, host and distribution: Described a new Genus Acariscus of the American Chiggers
Brennan 1946 [140]	Pine squirrels, mantled ground squirrels, white-footed mice, wood rats, woodchucks, pika	Montana and Idaho	*Chatia*	Taxonomy and host: Report of a new genus and species of chiggers
Ewing 1946 [48]	NA	NA	*Speotrombicula*	Chigger Taxonomy: Provided a key to chigger subfamilies with redescriptions of two old genera and descriptions of a new subfamily and a new genus
Jenkins 1947 [34]	NA	NA	*Eutrombicula alfreddugesi* and *E. masoni* and *E. batatas*	Chigger rearing methods
Doetschman and Furman 1949 [141]	Humans	California	*Eutrombicula batatas*	Report of chiggers attacking man in California
Jenkins 1949 [142]	Humans	NA	*Eutrombicula splendens*	Taxonomy, geographic distribution, and hosts of Eutrombicula splendens
Brennan and Wharton 1950 [143]	NA	NA	Subgenus *Neotrombicula*	Systematics, geographic distribution, seasonal occurrence, and host relations of the most common chiggers; provided key to species
Lipovsky and Loomis 1954 [144]	NA	Kansas, Oklahoma and Missouri	*Euschongastia jonesi*	Taxonomy: described a new species of chiggers
Crossley 1955 [145]	Colorado wood rats	Colorado	*Euschongastia finleyi*	Taxonomy: described a new species of chiggers
Hyland 1956 [146]	Southern leopard frog	Florida	*Hannemania hegeneri*	Taxonomy: described a new species of chiggers
Lipovsky et al. 1957 [16]	NA	NA	*Eutrombicula splendens* and *Hannemania* sp.	The mode of insemination of chiggers
Brennan and White 1960 [147]	Bats	Alabama	*Perissopalla flagellisetula*, *Euschongastia staffordi*, *Trombicula tibbettsi*, *Euschongastia pipistrelli*, *Euschongastia hamiltoni*, *Eutrombicula alfreddugesi*,	Taxonomy: descriptions of chiggers on bats in Alabama

			Eutrombicula batatas, *Trombicula gurneyi* and *Whartonia senase*	
Johnston and DeGiusti 1961 [148]	NA	Michigan and Ontario	*Gahrliepia (Walchia) americana*, *Leptotrombidium myotis*, *Miyatrombicula esoensis*, *Trombicula gurneyi*, *Euschongastia blarinae* and *Euschongastia crateris*	Taxonomy and Ecology: descriptions of collections of chiggers from soil and leaf litter.
Powder and Loomis 1962 [149]	Reptiles	Southern California	Odontacarus arizonensis, Odontacarus shawi, Eutrombicula belkini, Trombicula arenicola, Euschongastia lacerta and *Euschongastia longitarsala*	Survey of chiggers on reptiles
Loomis 1963 [150]	Desert Wood Rat, Canyon Mouse, Cactus mouse, Pinon mouse	Joshua Tree National Monument of California	*Microtrombicula nasalis* and *Microtrombicula wrenni*	Taxonomy: Report the discovery of two new species in the nasal passages of rodents from California
Brennan 1965 [151]	*Neotoma lepida*, *Pipistrellus hesperus*	Nevada and Arizona	*Euschoengastoides sloomi*, *Pseudoschoengastia aeci*, *Trombicula sprocssi*, *Trombicula univari* and *Trombicula veanda*	Taxonomy: Provided descriptions and figures of five new species from Southwestern United States
Brennan 1966 [152]	*Neotoma lepida*, *Peromyscus* sp.	California	*Odontacarus cognatus*, *Euschoengastoides ryckmani*, *Sasacarus whartoni*, *Euschoengastia lacerta*, *Leptotrombidium panamens*, *Trombicula allredi*, *Trombicula jessie*, *Trombicula lacerticol* and *Trombicula thompsoni*	Taxonomy: Provided descriptions of new chigger species in California
Johnston et al. 1966 [153]	Pilot black snake	Ohio	*Fonsecia palmella*	Taxonomy: Found an error in the original description of *Fonsecia palmella*
Webb and Loomis 1970 [154]	NA	NA	Subgenus *Crypticula*	Taxonomy: Describe a new subgenus of intranasal chiggers of the genus Microtrombicula from North America and Korea; provided a key to species
Crossley and Atyeo 1972 [155]	Nasal cavities of clapper rails	Alabama and Florida	*Blankaartia pauli*	Taxonomy: Describe a new intranasal species of chigger
Everett et al. 1972 [156]	Cardinal, House Sparrow,	Texas	*Neoschoengastia americana*	Hosts: Report of new host records of *Neoschoengastia americana* from Texas

	Mockingbird, Bobwhite, House Wren, Painted Bunting			
Easton 1975 [157]	Townsend's Chipmunk, Deer Mouse, Western Red-backed Mouse, Creeping Vole, Montane Vole, Dusky-footed Woodrat, Bushy- tailed Woodrat, Pacific Shrew, Vagrant Shrew and Trowbridge's Shrew	Western Oregon	*Euschoengastia oregonensis*, *Neotrombicula harperi*, *Comatacarus americanus*, *Neotrombiclla cavicola* and *Chatia setosa*	Hosts: Survey of chiggers on rodents in two forested areas of western Oregon
Brennan and Goff 1977 [64]	NA	NA	87 genera	Taxonomy: Provided keys to the genera of chiggers of the western hemisphere
Rohani and Cromroy 1979 [26]	Black racer, Corn snake, Eastern swift, Eastern grey squirrel, Box tortoise, Indigo snake, Eastern swift lizard, Common opossum, Barbour's pigmy rattlesnake, Yellow rat snake	Northcentral Florida	*Eutrombicula alfreddugesi*, *Eutrombicula splendens*, *Fonsecia (Parasecia) gurneyi gurneyi*, *Walchia americana*, *Euschongastia rubra*, *Miyatrombicula jonesae* and *Leptotrombidium peromysci*	Taxonomy and Distribution: Survey of chiggers from litter samples, tree holes, black plates, and vertebrate hosts in northcentral Florida; Provided a key to the chigger species collected and provided a list of the hosts of collected chiggers
Goff and Judd 1981 [158]	tortoise	Texas	*Eutrombicula alfreddugesi*	Report of a new host of *Eutrombicula alfreddugesi*

Pomeroy and Loomis 1984 [159]	NA	Western North America	*Euschoengastia furmani*, *Dermadelema mojavense* *D. sleeperi* and *D. lynnae*	Taxonomy: Proposed a new genus *Dermadelema* and described four species
Ludwig et al. 1985 [160]	Small mammals	Piedmont habitats of Georgia	*Eutrombicula alfreddugesi*	Host relationships and seasonal distribution
Durden and Wilson 1991 [161]	White-footed mice (woodland mice)	central Tennessee	*Comatacarus americanus*, *Euschoengastia peromysci*, and *Leptotrombidium peromysci*	Report of chiggers on rodents
Wilson et al. 1991 [162]	Urban Gray Squirrels	Northern Florida	*Eutrombicula alfreddugesi*, *E. splendens*, *Leptotrombidium peromysci* and *Parasecia gurneyi*	Report of chiggers on rodents
Durden 1992 [163]	Meadow voles	Maryland	Neotrombicula whartoni	Report of chiggers on rodents
Clopton and Gold 1993 [20]	NA	Nebraska	*Eutrombicula alfreddugesi*	Ecology: Geographical and seasonal distribution and diurnal activity patterns of chiggers in a forest edge ecosystem
Goldberg and Bursey 1993 [164]	lizard	Arizona	*Eutrombicula Iipovskyana*	Ecology: Duration of attachment on hosts
Anthony et al. 1994 [165]	Salamanders	Ouachita Mountains of Oklahoma and Arkansas	*Hannemania dunni*	Male salamanders have more chiggers than females.
Goldberg and Bursey 1994 [166]	lizard	Arizona	*Eutrombicula Iipovskyana*	Prevalence of chigger infestation on lizard
Pung et al. 1994 [167]	Opossums and Raccoons	Southeastern Georgia	*Eutrombicula alfreddugesi*, *Leptotrombidium peromysci*, and *Neotrombicula whartoni*	Survey of chiggers on rodents
Forrester et al. 1996 [168]	white-tailed deer	Southern Florida	*Eutrombicula splendens*	Survey of chiggers on deers
Durden et al. 1997 [169]	Eastern woodrats	South Carolina and Georgia	*Eutrombicula peromysci* and *Eutrombicula alfreddugesi*	Survey of chiggers on rodents
Goff and Mckown 1997 [170]	Domestic cat	Texas	*Hexidionis garfieldi*	Taxonomy: described a new species *Hexidionis garfieldi* collected from a cat, and given a key to the genus *Hexidionis*
Little et al. 1997 [171]	white-tailed deer	Georgia	Very similar to *Neotrombicula richmondi*	Reports of trombidiosis (dermatitis) in deers
Spalding et al. 1997 [172]	Sandhill cranes	Florida	*Blankaartia sinnamaryi*	Reports of trombidiosis (dermatitis) in sandhill cranes

Durden et al. 2000 [22]	Cotton mouse, Cotton rat, eastern woodrat, golden mouse, eastern gray squirrel	Northwestern Florida	*Cheladonta ouachitensis, Euschoengastia peromysci, Neotrombicula whartoni, Eutrombicula cinnabaris, Leptotrombidium peromysci*	Survey of chiggers on rodents
Pung et al. 2000 [173]	Southern flying squirrels	southeastern Georgia	*Leptotrombidium peromysci*	Survey of chiggers on rodents
Sladky et al. 2000 [174]	Tree frog	North Carolina Zoological Park	*Hannemani*a sp.	Report of chiggers on tree frogs
Williams et al. 2000 [175]	Northern Bobwhite Quail	Eastern Kansas	*Neotrombicula whartoni*	Report of chiggers on quails
Cunningham et al. 2001 [176]	Black bear	Florida	*Eutrombicula splendens*	Reports of trombidiosis (dermatitis) in black bear
Houck et al. 2001 [106]	White-footed mouse, marsh rice rat, fulvous harvest mouse, pigmy mouse, hispid cotton rat	Texas	*Eutrombicula splendens*	Provided the first evidence of hantavirus-specific RNA from *Eutrombicula splendens*
Durden et al. 2004 [56]	Gray squirrels	southeastern Georgia	*Eutrombicula splendens, Myiatrombicula cynos* and *Neotrombicula whartoni*	Survey of chiggers on rodents
Klukowski 2004 [177]	Eastern Fence Lizards	middle Tennessee	*Eutrombicula* sp.	Ecology: seasonal distribution of chiggers on lizards
Reeves et al. 2004 [40]	NA	South Carolina	*Comatacarus americanus, Euschoengastia blarinae, Euschoengastia jamesoni, Euschoengastia peromysci, Euschoengastia pipistrelle, Eutrombicula alfreddugesi, Eutrombicula splendens, Fonsecia palmella, Miyatrombicula cynos, Neoschoengastia americana, Neotrombicula autumnalis, Neotrombicula fitchi, Neotrombicula waynensis, Neotrombicula whartoni* and *Parasecia gurneyi*	Report 15 chigger species in South Carolina based on records from Clemson University Arthropod Collection and relevant literature

Malone and Paredes-Leon 2005 [178]	Cliff chirping frog	Texas	*Hannemania monticola*	Report of *Hannemania monticola* from frogs; the first record of *Hannemania monticola* in the US
Kurta et al. 2007 [179]	Mormoopid Bats	Puerto Rico	*Microtrombicula boneti, Microtrombicula carmenae, Perates discors, Perates monops, Whartonia guerrerensis*	Survey of chiggers on bats
Pearce and O'Shea 2007 [67]	Big brown bats	Colorado	*Leptotrombidium myotis*	Survey of chiggers on bats
Torrence et al. 2007 [180]	Two spadefoot toads	Texas	*Hannemania* sp.	Report of *Hannemania* sp. infestation on toads
Nims et al. 2008 [181]	Oldfield mice, eastern harvest mouse and short-tailed shrews	Southeastern Georgia	*Euschoengastia peromysci and Leptotrombidium Peromysci, Comatacarus americanus*	Surveys of chiggers on rodents
Westfall et al. 2008 [182]	Plethodontid salamanders	Western piedmont of North Carolina	*Hannemania dunni*	Patterns of *Hannemania dunni* parasitism among salamanders: comparison of chigger abundance from different boy locations of salamanders; evaluation of the relationship between salamander body size and chigger abundance, sample location and chigger abundance; examining seasonal variation in chigger abundance
Bulté et al. 2009 [183]	Yarrow's spiny lizards	Chiricahua mountains of southeastern Arizona	Not identified to genus	Report of chiggers on lizards
Mertins et al. 2009 [184]	Cattle egret	Florida Keys	*Whartonacarus floridensis* sp. nov.	Taxonomy: Described a new species in the genus *Whartonacarus*; provided a taxonomic review and revised key of the genus *Whartonacarus*
Corn et al. 2011 [185]	Burmese pythons	Florida	*Eutrombicula splendens* and *Eutrombicula cinnabaris*	Reports of chiggers from wild-caught exotic reptiles
Mertins et al. 2011 [52]	Spadefoot toads	Southern high plains of Texas	*Eutrombicula alfreddugesi*	Reexamined the chiggers reported from [66] and found the chiggers identified

				previously as *Hannemani*a sp. were *Eutrombicula alfreddugesi*
Walters et al. 2011 [17]	NA	NA	247 species (reported from the US)	Provided a list of chigger species, states and hosts reported from the US and Canada
McCoy et al. 2012 [186]	Florida sand skink and Florida scrub lizard, and the six-lined racerunner	Florida	*Eutrombicula* sp.	Ecology: Assessed the influence of fire histories on the prevalence of *Eutrombicula* sp. on lizards
McAllister et al. 2013 [57]	Ozark zig-zag salamanders	Northern Arkansas	*Hannemania dunni*	Survey of chiggers on salamanders
Bennett et al. 2014 [46]	NA	NA	*Eutrombicula cinnabaris*	Taxonomy: argued *Eutrombicula cinnabaris* should be the best available name for the species previously called *E. alfreddugesi*.
Crossley and Clement 2015 [45]	Rafinesque's Big-Eared Bat	Georgia	*Hoffmanniella solickiana*	Described the first record of one species in the genus *Hoffmanniella* from the US
Garvin et al. 2015 [53]	Lizards and snakes	Oklahoma	*Eutrombicula splendens and Eutrombicula cinnabaris*	Survey of chiggers on reptiles
McAllister et al. 2015 [58]	Southern Toad	Florida	*Hannemania hegeneri*	Survey of chiggers on toads
Ott-Conn et al. 2015 [187]	Amargosa vole	California	*Neotrombicula microti*	Report of chiggers on endangered Amargosa vole
McAllister et al. 2017 [188]	Rio Grande leopard frog and Plains leopard frog	Texas	*Hannemania dunni*	Survey of chiggers on amphibians
Milley et al. 2017 [189]	Community cats	Ohio	Not identified to genus	Survey of chiggers on cats
Bakkegard et al. 2019 [54]	Northern Slimy Salamanders	Alabama	*Hannemania dunni*	Survey of chiggers on salamanders
Harrity and Conway 2019 [190]	Yuma Ridgway's Rails	southwestern Arizona	*Blankaartia* spp.	Report of severe chigger infestation on an endangered bird species
Bassini-Silva et al. 2020 [191]	NA	NA	*Parasecia*	Ecology: provided a revision of the genus Parasecia and a key to species

Orton et al. 2020 [192]	Florida scrub lizards	Ocala National Forest (ONF) of central Florida	*Eutrombicula cinnabaris*	Relationship between sexual color in male Florida scrub lizards and chigger infestation
McAllister et al. 2021 [55]	Bats	Oklahoma	*Euschoengastia pipistrelli*	Provided some stereoscopic photographs of *Euschoengastia pipistrelli* infestation and a scanning electron micrograph of this chigger species; Provided a summary of host and state records for this chigger.
Nielsen et al. 2021 [5]	NA	NA	NA	Annotated world checklist of the Trombiculidae and Leeuwenhoekiidae (1758–2021)
McInnis et al. 2021 [193]	NA	NA	NA	Chiggers have been indicated in alpha-gal hypersensitivity.

* NA, not applicable.

Table S3. Number of chigger species reported in each US states. Data sources: Walters et al. 2011 [17], Crossley and Clement 2015 [45].

US states	Number of chigger species reported
Alabama	13
Alaska	3
Arizona	29
Arkansas	16
California	97
Colorado	30
Connecticut	1
Delaware	4
District Of Columbia	1
Florida	21
Georgia	21
Idaho	24
Illinois	11
Indiana	27
Iowa	2
Kansas	47
Kentucky	3
Louisiana	5
Maine	4
Maryland	11
Massachusetts	8
Michigan	15
Minnesota	4
Mississippi	6
Missouri	11
Montana	22
Nebraska	11
Nevada	30
New Hampshire	1
New Jersey	10
New Mexico	16

New York	14
North Carolina	22
North Dakota	2
Ohio	9
Oklahoma	21
Oregon	24
Pennsylvania	21
South Carolina	16
South Dakota	14
Tennessee	13
Texas	75
Utah	52
Vermont	2
Virginia	10
Washington	5
West Virginia	10
Wisconsin	6
Wyoming	5
Hawaii	0
